# Analysis for DC and RF Characteristics Recessed-Gate GaN MOSFET Using Stacked TiO_2_/Si_3_N_4_ Dual-Layer Insulator

**DOI:** 10.3390/ma15030819

**Published:** 2022-01-21

**Authors:** So-Ra Min, Min-Su Cho, Sang-Ho Lee, Jin Park, Hee-Dae An, Geon-Uk Kim, Young-Jun Yoon, Jae-Hwa Seo, Jae-Won Jang, Jin-Hyuk Bae, Sin-Hyung Lee, In-Man Kang

**Affiliations:** 1 School of Electronic and Electrical Engineering, Kyungpook National University, Daegu 41566, Korea; minsora0716@gmail.com (S.-R.M.); chominsu14@knu.ac.kr (M.-S.C.); jim782jim@naver.com (S.-H.L.); jdefs12@naver.com (J.P.); gmleo3396@naver.com (H.-D.A.); kku563@naver.com (G.-U.K.); j1jang@knu.ac.kr (J.-W.J.); jhbae@ee.knu.ac.kr (J.-H.B.); sinhlee@knu.ac.kr (S.-H.L.); 2 Korea Multi-Purpose Accelerator Complex, Korea Atomic Energy Research Institute, Gyeongju 38180, Korea; yjyoon@kaeri.re.kr; 3 Power Semiconductor Research Center, Korea Electrotechnology Research Institute, Changwon 51543, Korea; seojeahwa@naver.com

**Keywords:** gallium nitride (GaN), aluminum gallium nitride (AlGaN), self-heating effect (SHE), dual-layer insulator, silicon nitride (Si_3_N_4_), titanium dioxide (TiO_2_), sapphire, silicon carbide (SiC), radio frequency (RF)

## Abstract

The self-heating effects (SHEs) on the electrical characteristics of the GaN MOSFETs with a stacked TiO_2_/Si_3_N_4_ dual-layer insulator are investigated by using rigorous TCAD simulations. To accurately analyze them, the GaN MOSFETs with Si_3_N_4_ single-layer insulator are conducted to the simulation works together. The stacked TiO_2_/Si_3_N_4_ GaN MOSFET has a maximum on-state current of 743.8 mA/mm, which is the improved value due to the larger oxide capacitance of TiO_2_/Si_3_N_4_ than that of a Si_3_N_4_ single-layer insulator. However, the electrical field and current density increased by the stacked TiO_2_/Si_3_N_4_ layers make the device’s temperature higher. That results in the degradation of the device’s performance. We simulated and analyzed the operation mechanisms of the GaN MOSFETs modulated by the SHEs in view of high-power and high-frequency characteristics. The maximum temperature inside the device was increased to 409.89 K by the SHEs. In this case, the stacked TiO_2_/Si_3_N_4_-based GaN MOSFETs had 25%-lower values for both the maximum on-state current and the maximum transconductance compared with the device where SHEs did not occur; *R*_on_ increased from 1.41 mΩ·cm^2^ to 2.56 mΩ·cm^2^, and the cut-off frequency was reduced by 26% from 5.45 GHz. Although the performance of the stacked TiO_2_/Si_3_N_4_-based GaN MOSFET is degraded by SHEs, it shows superior electrical performance than GaN MOSFETs with Si_3_N_4_ single-layer insulator.

## 1. Introduction

Silicon (Si) is widely used in the semiconductor industry as it is a material with very stable physical properties. However, recognizing the band gap limit, the research on compound semiconductors such as gallium nitride (GaN) that can be used stably at high voltage and high frequency is considered to be an important topic [1,2,3]. The AlGaN/GaN-based high-electron-mobility transistor (HEMT) is suitable for power switching applications. The two-dimensional electron gas (2DEG) formed between AlGaN and GaN layers results in a high switching speed, low on-resistance, large current handling capabilities, and high breakdown voltage [4,5]. In addition, it has long been established as a promising candidate for high-frequency operation because the high saturation velocity of the electrons significantly enhances the transport properties [6]. Overall, although HEMT devices operate in enhancement-mode, the normally off operation is more appropriate for GaN-based transistors to target high-voltage power switching applications for fail-safe requirements and to simplify the design of driving circuits. Methods for normally off operations include gate-recess etching, fluorine plasma ion implantation, the p-type doped gate structure, and the gate-controlled tunnel junction, which has been proven to be capable of normally off operations [7,8,9,10]. Furthermore, HEMTs with a thin gate-insulator have a suppressed leakage current and high reliability due to their improved interface quality [11,12].

However, when the GaN devices operate at a high voltage region, the self-generated heat lowers the maximum power density and accelerates device failure [13]. The self-heating effects (SHEs) cause phonon scattering by increasing channel temperature, which limits the overall performance such as breakdown voltage, gate-leakage current, stability, and negatively sloped saturation curve. Thus, it is important to investigate and analyze models related to thermal behavior [14,15].

In our previous study, we compared the DC characteristics of recessed-gate MIS-HEMTs based on the variation of the Si_3_N_4_, TiO_2_ insulator thickness and demonstrated that the application of an appropriate combination of two materials improves the device’s DC electrical characteristics [16]. Further, detailed adjustments to the simulation parameters and models were performed, and it was examined that the results were observed to be the same in general. Although SHEs were applied, a thorough investigation of heat generation and RF properties for use in power amplifier applications were not included.

In conclusion, in this study, we compare the DC performance changes in GaN MOSFET using the stacked TiO_2_/Si_3_N_4_ dual- or Si_3_N_4_ single-layer insulator depending on whether the SHEs is applied and analyze the operation at a large RF frequency, which is expected to change due to dispersion of temperature, considering the thermal mechanism. In terms of SHEs, the most critical factor in determining the level of thermal rise inside a device is the thermal conductivity of each material used for device fabrication. When a device is made of a material with high thermal conductivity, heat generated spontaneously during operation can easily escape to the outside, which suppresses the device’s performance degradation [17]. Therefore, for the proposed device structure, we also experiment to explore the tendency and sensitivity of the performance change when materials with different thermal conductivities are used as substrates.

## 2. Materials and Methods

**Figure 1:**
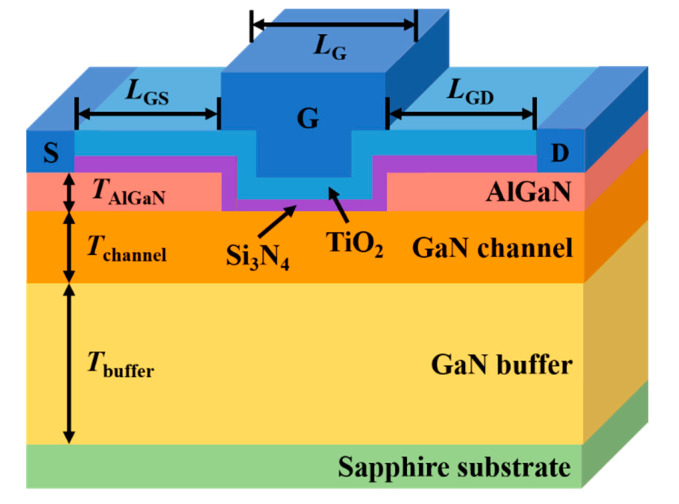
Schematic cross-section of a recessed-gate GaN MOSFET based on AlGaN/GaN heterostructure with TiO_2_ and Si_3_N_4_ as dual-layer insulator.

Figure 1 shows the cross-section of GaN MOSFET based on AlGaN/GaN heterostructure with a dual-layer insulator comprising Si_3_N_4_/TiO_2_ (10/20 nm thickness) under the recessed gate. The single-layer insulator in the device compared with the proposed structure has only a 30 nm thick Si_3_N_4_, and all other details and conditions are the same. The length of the gate head (*L*_G_) is 2 μm and the length from the gate to the source and drain is 5 μm each (*L*_GS_ and *L*_GD_, respectively), which are symmetrical in structure. The AlGaN layer is 25 nm thick on both sides under the insulator (*T*_AlGaN_), and the thickness of the GaN channel is 100 nm (*T*_channel_); GaN and AlGaN also form a 2DEG layer based on the heterostructure. The 2DEG layer under the gate is removed because there is a recessed gate with a 25 nm depth in the center, implying that the channels that were naturally created by the two materials are not formed. Thus, it operates similarly to a MOSFET that forms a channel when a positive voltage is applied, and the normally off operation is possible by shifting the threshold voltage in the positive direction. GaN buffer layer thickness is 2 μm (*T*_buffer_) under the channel and sapphire is used as a substrate.

Many studies have been conducted to determine an optimum substrate material and thickness to prevent instability and controllability degradation due to the temperature rise in the device. The method of changing the substrate does not require complicated procedures, is nondestructive, and simple to apply to existing technologies. Diamond, silicon carbide (SiC), Si, and sapphire have been compared as candidates for GaN substrates. Particularly, SiC and diamond, which have low thermal resistance when used with GaN, are suitable materials that can reduce the maximum temperature [18]. Table 1 shows that the thermal conductivity of sapphire and SiC is 35 and 420 W/mK, respectively, meaning SiC is more than 10 times higher; thus, the characteristic change can be confirmed due to the difference in temperature distribution and heat circulation. Therefore, we additionally confirm the electrical properties and RF performance variation when the substrate material was changed from sapphire to SiC with different thermal conductivity.

In this study, various models were applied to include the phenomena that occur during the operation of the device through the ATLAS technology computer-aided design simulation (Silvaco Inc., Santa Clara, CA, USA). Considering the piezoelectric and spontaneous polarization in the 2DEG layer between AlGaN and GaN, the strain due to lattice mismatch was automatically calculated and Shockley–Read–Hall recombination was applied as a physical model. In addition, the device’s DC characteristics were derived by adjusting the low and high field mobilities, and we obtained more accurate results by providing interface trap, thermal conductivity, impact ionization, lattice temperature, and permittivity values for each material.

When the device is turned on, a model that spontaneously increases the temperature is used, and this heat generation is explained by lattice heat flow and general thermal environments in the simulation. The equation for calculating the mechanism that changes due to heat generated by the SHEs can be expressed as follows:

(1) $$C\frac{{dT}_{L}}{dt}=\nabla (k\nabla T_{L})+H$$

where *C* is the heat capacitance per unit volume, *T*_L_ is the local lattice temperature, *k* is the thermal conductivity, and *H* is the heat generation. The peak of temperature and the temperature distribution are calculated and determined through numerical simulation when the temperature of the lattice increases by applying bias. This model calculates the lattice temperature depending on the material and transmission parameters. It also supports general thermal environment specifications using a combination of realistic heat sink construction, thermal impedance, and specified ambient temperature [23,24,25,26]. In addition, for heat flow, the Neumann boundary condition is set as a default value at all boundaries except for the floor, if the model that controls the movement of heat to the floor is not applied; thus, we must provide the thermal resistance values to aid our calculations. Because the thermal contact is not set on the top to focus on the bottom, which is the path where heat escapes to the outside, the movement of heat in the device is determined to be in the direction of the bottom [27].

## 3. Results

### 3.1. Dependence of Heat Generation on Oxide Capacitance

Although SiO_2_ as a gate-insulator material can prevent leakage current, it has low transconductance (*g*_m_) and large pinch-off voltage, which causes many problems in terms of scaling the device down. Alternatively, high-k dielectrics, such as TiO_2_, Al_2_O_3_, and HfO_2_, minimize gate-leakage current, increase transconductance, and have high breakdown voltages, making it possible to have a performance suitable for power devices [28,29]. TiO_2_ and Si_3_N_4_, which we adopted as high-k gate-insulator materials, have dielectric constants of 80 and 8, respectively. When TiO_2_ is used alone as a gate-insulator, although the high dielectric constant can result in better electrical properties, its small band gap generates a large leakage current compared with other high-k materials. Furthermore, sputtering deposition directly on GaN produces poor quality that loses the function of the insulator to prevent leakage. Therefore, it is possible to maintain a high capacitance value by stacking TiO_2_ on Si_3_N, and Si_3_N_4_ is already frequently used for passivation of GaN devices, so it can solve the difficulties in the process. Deposition is possible through various methods such as in situ deposition in the metalorganic chemical vapor deposition (MOCVD) chamber, plasma-enhanced chemical vapor deposition (PECVD), and low-pressure chemical vapor deposition (LPCVD) [30]. The formula for calculating the capacitance of an insulator composed of two materials is as follows:

(2) $$C_{{\mathrm{TiO}}_{2}}=\frac{\varepsilon_{{\mathrm{TiO}}_{2}}\;\cdot {\;\varepsilon }_{0}}{t_{{\mathrm{TiO}}_{2}}}$$

(3) $$C_{{\mathrm{Si}}_{3}N_{4}}=\frac{\varepsilon_{{\mathrm{Si}}_{3}N_{4}\;}\cdot {\;\varepsilon }_{0}}{t_{{\mathrm{Si}}_{3}N_{4}}}$$

(4) $$\frac{1}{C_{\mathrm{total}}}=\frac{1}{C_{{\mathrm{Si}}_{3}N_{4}}}+\frac{1}{C_{{\mathrm{TiO}}_{2}}}$$

where $\varepsilon_{{\mathrm{TiO}}_{2}}$ and $\varepsilon_{{\mathrm{Si}}_{3}N_{4}\;}$ are relative dielectric constants ($\varepsilon_{{\mathrm{TiO}}_{2}}$ = 80, $\varepsilon_{{\mathrm{Si}}_{3}N_{4}\;}$ = 8) of TiO_2_ and Si_3_N_4_, respectively; $\varepsilon_{0}$ is the vacuum permittivity. Using $t_{{\mathrm{TiO}}_{2}}$ and $t_{{\mathrm{Si}}_{3}N_{4}}$ as the thicknesses of TiO_2_ and Si_3_N_4_ ($t_{{\mathrm{TiO}}_{2}}$ = 20 nm, $t_{{\mathrm{Si}}_{3}N_{4}}\;$ = 10 nm), respectively, $C_{{\mathrm{TiO}}_{2}}$ and $C_{{\mathrm{Si}}_{3}N_{4}}\;$ can be calculated; in the case of a dual-layer insulator the accumulation capacitance ($C_{\mathrm{total}}$) can be calculated from Equation (4), considering that the capacitors are connected in series. The calculated value of $C_{{\mathrm{Si}}_{3}N_{4}}\;$ in the device with a single-layer insulator is 236 nF/cm^2^, whereas the $C_{\mathrm{total}}\;$ in the device using a dual-layer insulator is about 590 nF/cm^2^, which is roughly twice as large, implying superior current characteristics. Moreover, it shows that a high capacitance value can be induced under the condition that the ratio of $t_{{\mathrm{TiO}}_{2}}\;$ is greater than $t_{{\mathrm{Si}}_{3}N_{4}}\;$ for a constant insulator thickness of 30 nm.

**Figure 2:**
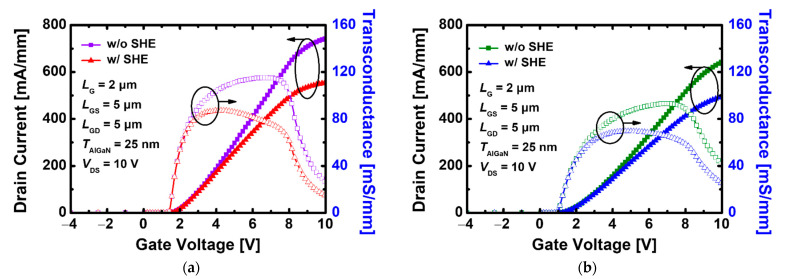
*I*_D_-*V*_G_ transfer characteristics with and without SHE in recessed-gate GaN MOSFET using (**a**) the stacked TiO_2_/Si_3_N_4_ dual-layer insulator, (**b**) Si_3_N_4_ single-layer insulator at *V*_DS_ = 10 V.

Figure 2a,b shows the drain current (*I*_D_)-gate voltage (*V*_G_) transfer curves when SHE is applied and not applied to recessed-gate GaN MOSFET devices with the stacked TiO_2_/Si_3_N_4_ dual- and Si_3_N_4_ single-layer insulators, respectively. Without the SHE, when *V*_GS_ is applied from 4 to 10 V, the maximum *I*_D_ (*I*_D, max_) in the device using the stacked TiO_2_/Si_3_N_4_ dual-layer insulator at bias *V*_DS_ = 10 V is 743.80 mA/mm, which is over 15% higher than 643.98 mA/mm of the device with the Si_3_N_4_ single-layer insulator and their maximum transconductance (*g*_m, max_) are 115.19 and 93.06 mS/mm, respectively. With the SHE, under the same conditions, *I*_D, max_ is 555.15 mA/mm when a stacked TiO_2_/Si_3_N_4_ dual-layer insulator is used, which is 12% higher than 495.61 mA/mm of the device with a Si_3_N_4_ single-layer insulator, and *g*_m, max_ is 87.30, 69.55 mS/mm, respectively. In Figure 2a,b, the *V*_G_ with the maximum value of transconductance moves in the negative direction under the influence of the SHE, confirming the tendency that the devices with the stacked TiO_2_/Si_3_N_4_ dual-layer insulators have a larger value than devices with a Si_3_N_4_ single-layer insulator for both characteristics, whether the SHE is applied. As aforementioned, the capacitance value connected in series due to TiO_2_ and Si_3_N_4_ is much larger than when only Si_3_N_4_ is used, and this is a significant factor that has a great influence on the current increase despite the performance degradation caused by SHEs.

Before we analyze the effect of heat in this study based on the type and thickness of the material used as a gate insulator, it is necessary to first understand how the device’s self-heating system works. In Silvaco ATLAS, the overall mechanism that changes due to heat generation and heat flow is calculated from the joule heating, and the equation is as follows:

(5) $$H=(\;\vec{J_{n}}+\vec{J_{p}}\;)\;\cdot \;E$$

where *H* represents the generated heat, $\vec{J_{n}}$ and $\vec{J_{p}}$ represent the electron and hole current density, respectively, and *E* denotes the electric field. The heat generation in the GaN channel, which depends on the current density and electric field, contributes to the determination of the electrical properties according to insulator type; thus, the analysis of the correlation between these two factors is required.

**Figure 3:**
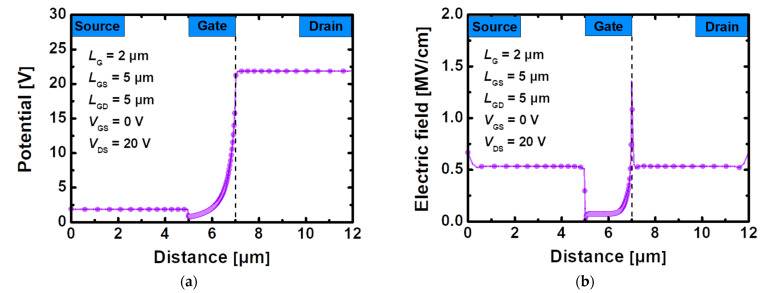
(**a**) Potential and (**b**) electric field in 2DEG layer between GaN and AlGaN under bias *V*_GS_ = 0 V, *V*_DS_ = 20 V for recessed-gate GaN MOSFET with the stacked TiO_2_/Si_3_N_4_ dual-layer insulator.

Figure 3a,b can play an auxiliary role in the convenient visualization of theoretical content. Figure 3a shows the overall potential distribution for the entire region in the device using the stacked TiO_2_/Si_3_N_4_ dual-layer insulator when a horizontal cutline is drawn along the channel where the 2DEG exists. In the off state (*V*_GS_ = 0 V) with *V*_DS_ applied to 20 V, an abrupt change occurs in the drain-side gate edge region, resulting in a large voltage drop. Figure 3b shows that the electric field is close to 0 due to the presence of Si_3_N_4_ instead of AlGaN under the recessed gate, and a distribution with a tendency similar to the potential that rapidly rises at the gate edge of the drain side is also shown. This means that it can withstand a strong electric field with the voltage drop which implies that it generates most of the heat and has the highest temperature value in this region [31].

### 3.2. Temperature Sensitivity Comparison

**Figure 4:**
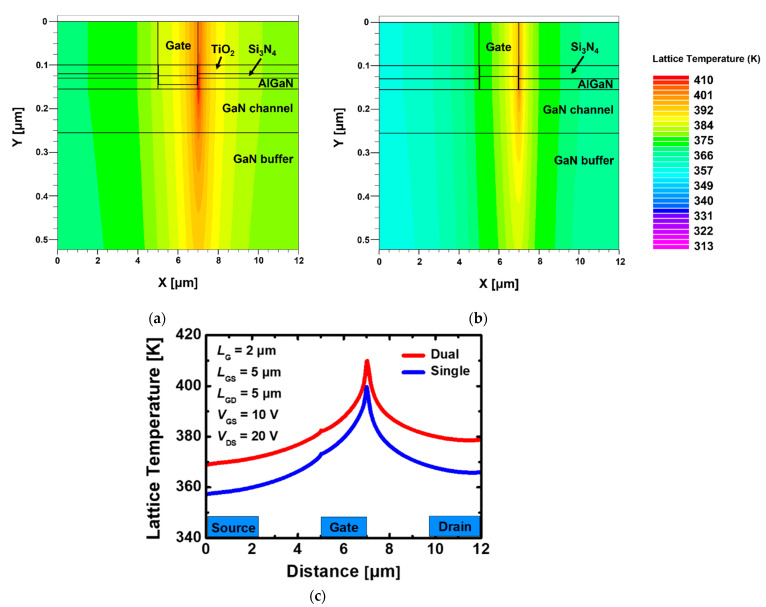
Cross-section of lattice temperature distribution in recessed-gate GaN MOSFET using (**a**) the stacked TiO_2_/Si_3_N_4_ dual-layer insulator, (**b**) Si_3_N_4_ single-layer insulator and (**c**) lattice temperature distribution according to AlGaN/GaN interface and channel layer for (**a**,**b**) when self-heating effect is applied at *V*_DS_ = 20 V, *V*_GS_ = 10 V.

**Figure 5:**
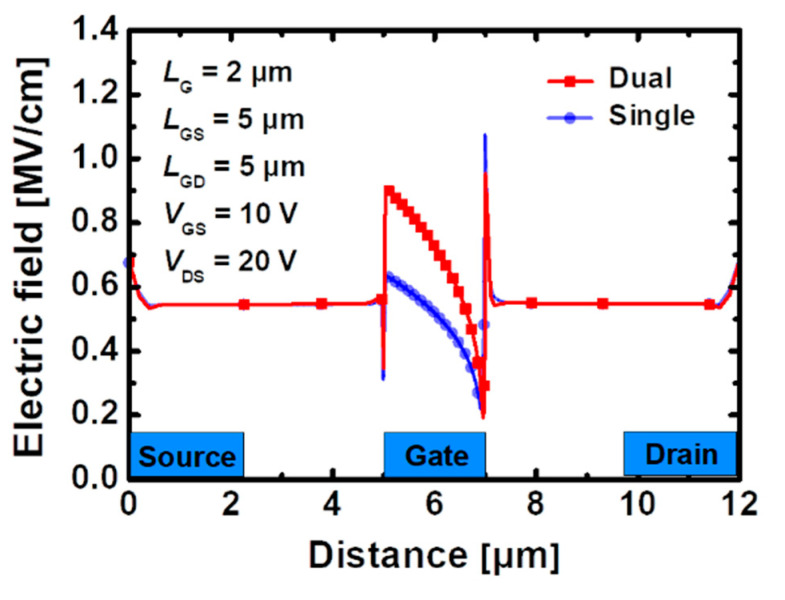
Electric field distribution across the 2-DEG channel layer when self-heating effect is applied in recessed-gate GaN MOSFET using the stacked TiO_2_/Si_3_N_4_ dual-layer insulator and Si_3_N_4_ single-layer insulator under bias *V*_GS_ = 10 V, *V*_DS_ = 20 V.

Figure 4a,b show the lattice temperature distribution in a device with the stacked TiO_2_/Si_3_N_4_ dual- and Si_3_N_4_ single-layer insulators, in which heat flow is reflected and particle motions, such as mobility and scattering, are calculated under the bias *V*_DS_ = 20 V and *V*_GS_ = 10 V. In both cases, it indicates that the hottest part where the heat is concentrated is the drain-side gate edge region. The peak temperature in the device using the stacked TiO_2_/Si_3_N_4_ dual-layer insulator with SHE is 409.89 K, whereas the device using the Si_3_N_4_ single-layer insulator rises to 398.75 K, which is 2.7% smaller according to Figure 4c. The field is formed relatively higher along the channel when the stacked TiO_2_/Si_3_N_4_ dual-layer insulator is used, whereas the gate-edge portion where the strongest electric field is generated appears to be larger in the device using the Si_3_N_4_ single-layer insulator as shown in Figure 5. However, there is a more pronounced difference in the current density compared with the electric field for the two types of devices. Therefore, the maximum temperature at the hotspot is higher in GaN MOSFET with the stacked TiO_2_/Si_3_N_4_ dual-layer insulator and the current density is much larger.

**Figure 6:**
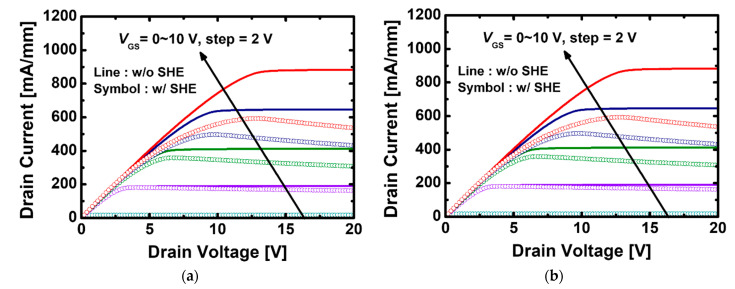
*I*_D_-*V*_D_ transfer characteristics with and without self-heating effect in recessed-gate GaN MOSFET using (**a**) the stacked TiO_2_/Si_3_N_4_ dual-layer insulator, (**b**) Si_3_N_4_ single-layer insulator.

Figure 6a,b shows the *I*_D_-drain voltage (*V*_D_) transfer curves for two types of devices with and without SHE. In the saturation region, as the slope of the curve decreases and the current tends to be constant, when SHE is not applied, whereas the saturation current is degraded when SHE is applied [32]. This phenomenon is because the increasing electric field and current density contribute to heat generation, and as thermal scattering is accelerated, electron mobility is reduced. When *V*_GS_ = 10 V, *I*_D, max_ is 883.05 mA/mm for GaN MOSFET with the stacked TiO_2_/Si_3_N_4_ dual-layer insulator without SHE, and the *I*_D_ decreases to 536.20 mA/mm when the heat is generated. Furthermore, *I*_D, max_ of GaN MOSFET with a Si_3_N_4_ single-layer insulator is 727.71 and 463.98 mA/mm with and without SHE, respectively, and it is confirmed that the electrical performance is lowered by SHE. The lattice has a relatively larger peak temperature value when the stacked TiO_2_/Si_3_N_4_ dual-layer insulator is used; however, it still has a higher *I*_D_ despite severe thermal scattering because the current density is significantly higher in an environment with a constant heat of 300 K. This implies that if the characteristics of both devices are analyzed at the same temperature, additional benefits in terms of current can be obtained when the dual-layer insulator is used.

**Figure 7:**
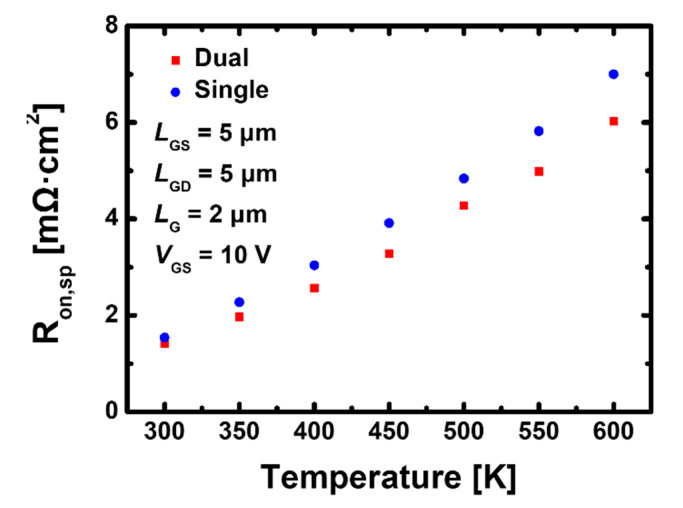
Specific on resistance according to internal temperature obtained from *I*_D_-*V*_D_ curve in GaN device using the stacked TiO_2_/Si_3_N_4_ dual-layer insulator and Si_3_N_4_ single-layer insulator.

Figure 7 shows the specific on resistance value extracted from the *I*_D_-*V*_D_ transfer curve when the lattice temperature is increased from 300 K to 600 K to examine the change trend of electrical characteristics under the same lattice temperature. The self-heating model produces temperature dispersion, but in this experiment, the temperature in all regions was set to be the constant. The resistance is calculated from the following equation:

*R*_on, sp_ = *R*_on_ ∙ *W* ∙ *L*_SD_ (6)

where *R*_on_ is on resistance value when a model that sets the constant to 300–600 K is applied under bias *V*_GS_ = 10 V, *W* is the width of the device, and *L*_SD_ is the length from the source to the drain [33]. The difference in resistance for two devices is due to the change in the insulator type, and considering that the parasitic resistance is the same, we can expect that the *R*_channel_ dominates. Regardless of the type of gate-insulator, the values of *R*_on, sp_ tend to increase linearly with temperature increases. At 300 K, the resistance of the device with the stacked TiO_2_/Si_3_N_4_ dual-layer insulator is 1.42 mΩ·cm^2^, which is about 7.8% smaller than the resistance value of 1.54 mΩ·cm^2^ for the device with a Si_3_N_4_ single-layer insulator and the resistance values of the GaN MOSFET with the stacked TiO_2_/Si_3_N_4_ dual- and Si_3_N_4_ single-layer insulators are 6.02 and 7.00 mΩ·cm^2^, respectively, which is about 16% larger, at 600 K.

Moreover, a Si_3_N_4_ single-layer insulator makes the slope of resistance steeper with increasing temperature. At high temperatures, the *R*_on, sp_ of the recessed-gate GaN MOSFET using the stacked TiO_2_/Si_3_N_4_ dual-layer insulator maintains a smaller value than that using the Si_3_N_4_ single-layer insulator; thus, it is estimated that carrier movement in the channel will be easier.

**Figure 8:**
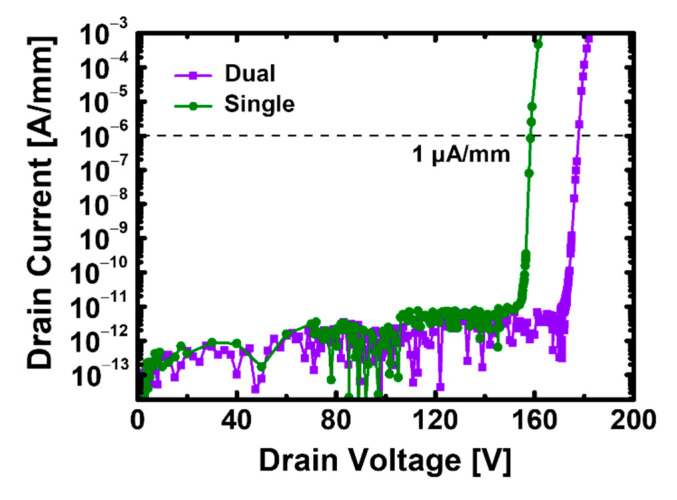
Breakdown voltage characteristics for recessed-gate GaN MOSFET based on AlGaN/GaN heterostructure with the stacked TiO_2_/Si_3_N_4_ dual-layer insulator and Si_3_N_4_ single-layer insulator at *V*_GS_ = 0 V.

Figure 8 shows the breakdown voltage characteristics of the recessed-gate GaN MOSFET when the stacked TiO_2_/Si_3_N_4_ dual- and Si_3_N_4_ single-layer insulators are used. We set the voltage at which the *I*_D_ = 1 μA/mm as the breakdown voltage and observed the breakdown voltage of the device composed of Si_3_N_4_ and TiO_2_ and that composed of Si_3_N_4_ to be 178 and 158 V, respectively. The device with the stacked TiO_2_/Si_3_N_4_ dual-layer insulator had a larger breakdown due to the effective dispersion when a high voltage is applied; thus, it has a stronger ability to withstand the high heat generated by the high voltage from a device operation perspective [16].

**Figure 9:**
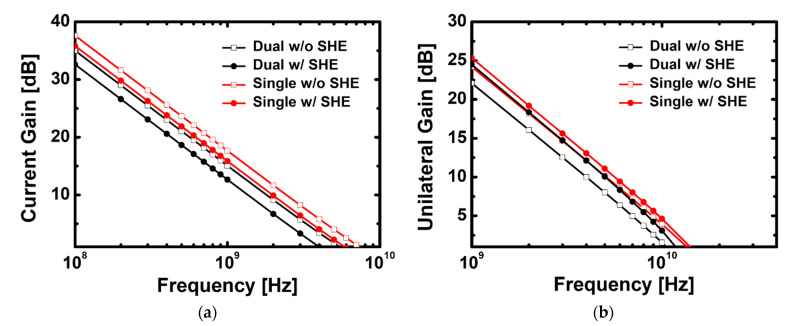

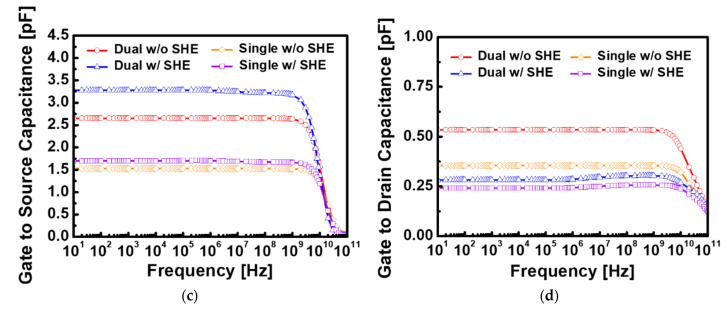
(**a**) Cut-off frequency, (**b**) maximum frequency and dependence of (**c**) gate to source capacitance and (**d**) gate to drain on frequency for the recessed-gate GaN MOSFET using the stacked TiO_2_/Si_3_N_4_ dual-layer insulator and Si_3_N_4_ single-layer insulator with and without self-heating effect.

Figure 9a,b shows the current and unilateral gains based on frequency increase in the recessed-gate GaN MOSFET with the stacked TiO_2_/Si_3_N_4_ dual- and Si_3_N_4_ single-layer insulators, where cut-off frequency (*f*_T_) and maximum oscillation frequency (*f*_max_) values were extracted at high frequency with and without SHE. The RF characteristics analysis is possible using the equation derived from the Y-parameter, and the equation is as follows:

(7) $$f_{T}=\frac{g_{m}}{{2\pi \;(C}_{\mathrm{gs}}{+C}_{\mathrm{gd}})}$$

(8) $$f_{\max}=\frac{f_{T}}{\sqrt{{4R}_{g}\;\cdot {\;(g}_{\mathrm{ds}}{+2\pi f}_{T}C_{\mathrm{gd}})}}$$

where *g*_m_ represents the transconductance, *C*_gs_ and *C*_gd_ are the extrinsic gate–source and gate–drain capacitance (expressed as *C*_ox_ = *C*_gs_ + *C*_gd_), respectively, *R*_g_ is the gate resistance, and *g*_ds_ is source-drain conductance. From the *I*_D_-*V*_G_ transfer characteristics curve, the *V*_G_ at which *g*_m_ becomes maximum for each case was applied on the RF simulation. It was induced by increasing transconductance and oxide capacitance, since GaN MOSFET with the stacked TiO_2_/Si_3_N_4_ dual-layer insulator aims to improve the DC characteristics; but Equation (5) shows that *g*_m_ and *C*_ox_ for *f*_T_ have opposite relationships, requiring a complex analysis. Table 2 summarizes the capacitance values and *f*_T_ calculated using the Y-parameter extracted by applying the AC signal model to each case. The cut-off frequency has a larger value when a Si_3_N_4_ single-layer insulator is used, and the SHE is not applied for GaN MOSFET as shown in Figure 9a.

Because *V*_D_ and *V*_G_ are changed by AC signals, unlike in DC signals, which affect the charge of the channel and gate, the *C*_gd_ and *C*_gs_ should be separately analyzed; thus, the semiconductor oxide capacitance value can no longer be defined as only *C*_ox_. Figure 9c,d shows *C*_gd_ and *C*_gs_ in the frequency range of 10–10^11^ Hz for the four cases in Figure 9a,b, where *C*_gs_ is dominant in determining the oxide capacitance value. The *C*_gs_ is larger for devices with SHE than those without SHE, which is due to a decrease in the thermally activated carriers’ detrapping phenomena in the donor layer, resulting in a decrease in the equivalent doping level; thus, smaller *C*_gs_ is observed at lower temperatures [34]. We confirm from this capacitance analysis that the *C*_gs_ of devices with a stacked TiO_2_/Si_3_N_4_ dual-layer insulator is large, as *g*_m_ has a significantly large value, whereas the *g*_m_ of devices with a Si_3_N_4_ single-layer insulator is relatively small. However, higher frequency operations in terms of RF characteristics are possible since *C*_gs_ is extremely small. The *f*_max_ to which the SHE is applied has a larger value than that to which the SHE is not applied in the two types of devices, unlike Figure 9a. Table 2 shows that the values of *C*_gs_ and *C*_gd_ are reversed based on whether SHE is applied because the value of the *V*_G_ in the section where *g*_m_ is the maximum decreases as the temperature increase; therefore, the capacitance value is estimated to be small when that value is applied during AC simulation, which affects these results. However, it is unchanged that the maximum frequency value is larger when GaN MOSFET is used as a Si_3_N_4_ single-layer insulator.

### 3.3. Heat Transfer Materials

**Figure 10:**
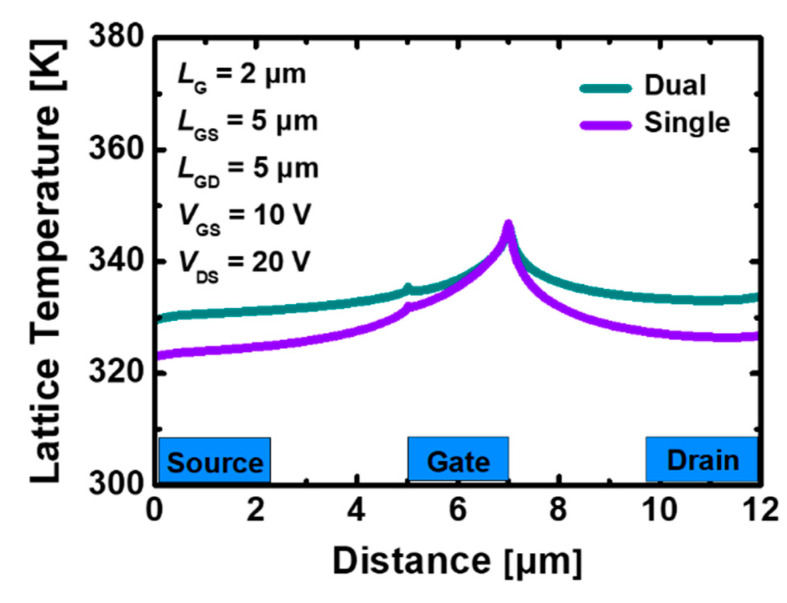
Lattice temperature distribution across the 2DEG channel layer with self-heating effect in recessed-gate GaN MOSFET using the stacked TiO_2_/Si_3_N_4_ dual-layer insulator and Si_3_N_4_ single-layer insulator on SiC substrate under bias *V*_GS_ = 10 V, *V*_DS_ = 20 V.

Figure 10 shows the distribution of the lattice temperature when only the substrate material is changed to SiC under the same device structure and bias conditions. The overall temperature difference relatively reduces and the peak temperature value at the drain side gate-edge of the GaN MOSFET using the stacked TiO_2_/Si_3_N_4_ dual- and Si_3_N_4_ single-layer insulators is 346.75 and 346.79 K, respectively; it exhibits an inverted trend compared to when the substrate is used as a sapphire. We demonstrate that SiC, which has excellent heat-transfer ability, can reduce the self-heating damage by preventing heat generated during device operation from being trapped inside and emitting it to the outside.

**Figure 11:**
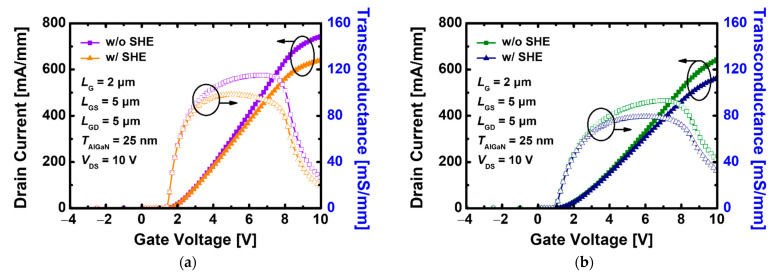
*I*_D_-*V*_G_ transfer characteristics with and without SHE in recessed-gate GaN MOSFET on SiC using (**a**) the stacked TiO_2_/Si_3_N_4_ dual-layer insulator, (**b**) Si_3_N_4_ single-layer insulator at *V*_DS_ = 10 V.

Because the replacement of the substrate material has an effect only when heat transfer occurs due to an increase in internal temperature, it does not affect the overall device properties while maintaining a constant 300 K without SHE. Figure 11a,b show that the use of SiC increases the *I*_D, max_ of the GaN MOSFET with the stacked TiO_2_/Si_3_N_4_ dual- and Si_3_N_4_ single-layer insulators to 640.52 and 564.29 mA/mm, respectively, and narrows the performance gap with the device without a temperature change.

**Figure 12:**
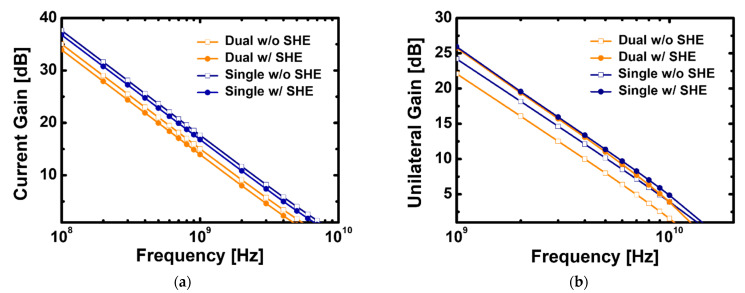
(**a**) Cut-off frequency and (**b**) maximum frequency of recessed-gate GaN MOSFET using the stacked TiO_2_/Si_3_N_4_ dual-layer insulator and Si_3_N_4_ single-layer insulator with and without self-heating effect on SiC substrate.

Figure 12a,b show the current and power gain based on the frequency obtained by applying the changed *V*_G_ at which *g*_m_ becomes maximum by changing the substrate to SiC. The RF characteristic remains constant regardless of the substrate material change without temperature rise. When SHE is applied, the *f*_T_ of devices with the stacked TiO_2_/Si_3_N_4_ dual- and Si_3_N_4_ single-layer insulators is 4.7 and 6.64 GHz, respectively, which are improved by 18% and 13% compared with GaN on the sapphire. Consequently, the difference from the frequency without SHE is also minimized, and *f*_max_ results are improved, reflecting the same trend.

## 4. Conclusions

We have analyzed the recessed-gate GaN MOSFET with the stacked TiO_2_/Si_3_N_4_ dual-layer insulator for several DC and RF characteristics by using a TCAD simulation. By increasing the oxide capacitance of the Si_3_N_4_ and TiO_2_ combination, we have confirmed that it has a smaller *R*_on_, larger *I*_D_ and improved *g*_m_ compared with the device using a Si_3_N_4_ single-layer insulator. The breakdown voltage is also relatively high, so it has strength as a power device. Furthermore, RF characteristics including current gain and power gain were evaluated. In addition, the self-heating effects (SHEs) model were reflected in the simulation, and important changes in DC and RF characteristics occurred. The performance degradation by SHEs is more affected for GaN MOSFETs with the stacked TiO_2_/Si_3_N_4_ dual-layer insulators due to its larger electric field and current density. Nevertheless, the dual-layer insulator induces the transistor to have enhanced DC performances. In conclusion, the recessed-gate GaN MOSFETs with the stacked TiO_2_/Si_3_N_4_ dual-layer insulator can be expected to be candidates for devices with an attractive ability to deliver high power at high frequency.

## Figures and Tables

**Table 1:** Thermal conductivity at 300 K of materials used in the proposed recessed-gate GaN MOSFET [19,20,21,22].

Material	Thermal Conductivity (W/mK)
Si_3_N_4_	30
TiO_2_	8.4
GaN	150
AlGaN	30
SiC	420
Sapphire	35

**Table 2:** Cut-off frequency, gate to source capacitance, gate to drain capacitance and gate oxide capacitance values calculated (*C*_ox_ = *C*_gs_ + *C*_gd_) according to whether SHE is applied or not in response to an AC signal is applied for recessed-gate GaN MOSFET with the stacked TiO_2_/Si_3_N_4_ dual- and Si_3_N_4_ single-layer insulators.

Material	*f*_T_ [GHz]	*C*_gs_ [pF]	*C*_gd_ [pF]	*C*_ox_ [pF]
Dual w/o SHE	5.45	2.57	0.53	3.10
Dual w/SHE	3.98	3.04	0.30	3.34
Single w/o SHE	7.36	1.51	0.35	1.86
Single w/SHE	5.86	1.65	0.26	1.91

## Data Availability

Not applicable.

## References

[1] Gupta C., Ji D., Chan S.H., Agarwal A., Leach W., Keller S., Chowdhury S., Mishra U.K. (2017). Impact of trench dimensions on the device performance of GaN vertical trench MOSFETs. IEEE Electron Device Lett..

[2] Ji D., Li W., Chowdhury S. (2018). A study on the impact of channel mobility on switching performance of vertical GaN MOSFETs. IEEE Trans. Electron Devices.

[3] Tsai C.Y., Wu T.L., Chin A. (2012). High-Performance GaN MOSFET with High-k LaAlO_3_/SiO_2_ Gate Dielectric. IEEE Electron Device Lett..

[4] Subramani N.K., Couvidat J., Al Hajjar A., Nallatamby J.C., Sommet R., Quéré R. (2017). Identification of GaN buffer traps in microwave power AlGaN/GaN HEMTs through low frequency S-parameters measurements and TCAD-based physical device simulations. IEEE J. Electron Devices Soc..

[5] Lee C.T., Chiou Y.L., Lee C.S. (2010). AlGaN/GaN MOS-HEMTs with gate ZnO dielectric layer. IEEE Electron Device Lett..

[6] Hirama K., Kasu M., Taniyasu Y. (2012). RF high-power operation of AlGaN/GaN HEMTs epitaxially grown on diamond. IEEE Electron Device Lett..

[7] Huang H., Liang Y.C., Samudra G.S., Ngo C.L.L. (2014). Au-free normally-off AlGaN/GaN-on-Si MIS-HEMTs using combined partially recessed and fluorinated trap-charge gate structures. IEEE Electron Device Lett..

[8] Tang Z., Jiang Q., Lu Y., Huang S., Yang S., Tang X., Chen K.J. (2013). 600-V Normally Off SiN_x_/AlGaN/GaN MIS-HEMT With Large Gate Swing and Low Current Collapse. IEEE Electron Device Lett..

[9] Oka T., Nozawa T. (2018). AlGaN/GaN recessed MIS-gate HFET with high-threshold-voltage normally-off operation for power electronics applications. IEEE Electron Device Lett..

[10] Yuan L., Chen H., Chen K.J. (2011). Normally off AlGaN/GaN metal–2DEG tunnel-junction field-effect transistors. IEEE Electron Device Lett..

[11] Bi Z., Hao Y., Liu H., Liu L., Feng Q. Characteristics analysis of gate dielectrics in AlGaN/GaN MIS-HEMT. Proceedings of the IEEE International Conference of Electron Devices and Solid-State Circuits (EDSSC).

[12] Huang Z., Liu J., Huang X., Yao J., Zhang J., Guo Y. Analysis of Interface Properties in AlGaN/GaN MIS-HEMTs with HfO_2_ and SiN_x_ Gate Dielectric. Proceedings of the 10th International Conference on Power and Energy Systems (ICPES).

[13] Jang K.W., Hwang I.T., Kim H.J., Lee S.H., Lim J.W., Kim H.S. (2020). Thermal analysis and operational characteristics of an AlGaN/GaN High electron mobility transistor with copper-filled structures: A simulation study. Micromachines.

[14] Sahoo A.K., Subramani N.K., Nallatamby J.C., Sommet R., Quéré R., Rolland N., Medjdoub F. Thermal analysis of AlN/GaN/AlGaN HEMTs grown on Si and SiC substrate through TCAD simulations and measurements. Proceedings of the 11th European Microwave Integrated Circuits Conference (EuMIC).

[15] Aouf A., Djeffal F., Douak F. Thermal stability investigation of power GaN HEMT including self-heating effects. Proceedings of the 6th International Conference on Systems and Control (ICSC).

[16] Jung J.H., Cho M.S., Jang W.D., Lee S.H., Jang J.W., Bae J.H., Kang I.M. (2020). Recessed-Gate GaN Metal-Insulator-Semiconductor High-Electron-Mobility Transistor Using a Dual Gate-Insulator Employing TiO_2_/SiN. J. Nanosci. Nanotechnol..

[17] Ranjan K., Arulkumaran S., Ng G.I., Sandupatla A. (2019). Investigation of Self-Heating Effect on DC and RF Performances in AlGaN/GaN HEMTs on CVD-Diamond. IEEE J. Quantum Electron.

[18] Podder A.K., Islam A.J., Hasanuzzaman S.M., Islam M.S., Bhuiyan A.G. Substrate effects on channel temperature distribution of AlGaN/GaN HEMT. Proceedings of the 3rd International Conference on Electrical Information and Communication Technology (EICT).

[19] Sarua A., Ji H., Hilton K.P., Wallis D.J., Uren M.J., Martin T.A.M.T., Kuball M. (2007). Thermal boundary resistance between GaN and substrate in AlGaN/GaN electronic devices. IEEE Trans. Electron Devices.

[20] Belkacemi K., Hocine R. (2018). Efficient 3D-TLM modeling and simulation for the thermal management of microwave AlGaN/GaN HEMT used in high power amplifiers SSPA. Int. J. Eng. Res. Appl..

[21] Tawfik M.M. (2017). Experimental studies of nanofluid thermal conductivity enhancement and applications: A review. Renew. Sustain. Energy Rev..

[22] Rutkowski P., Stobierski L., Górny G. (2014). Thermal stability and conductivity of hot-pressed Si 3 N 4–graphene composites. J. Therm. Anal. Calorim..

[23] Silvaco (2010). Atlas User’s Manual Device Simulation Software.

[24] Arivazhagan L., Jarndal A., Nirmal D. (2021). GaN HEMT on Si substrate with diamond heat spreader for high power applications. J. Comput. Electron.

[25] Orouji A.A., Rahimian M. (2011). Dual material insulator SOI-LDMOSFET: A novel device for self-heating effect improvement. Physica E Low Dimens. Syst. Nanostruct..

[26] Basumatary B., Maity S., Muchahary D. (2016). Improvement of drain current of AlGaN/GaN-HEMT through the modification of negative differential conductance (NDC), current collapse, self-heating and optimization of double hetero structure. Superlattices Microstruct..

[27] Vitanov S., Palankovski V., Maroldt S., Quay R. (2010). High-temperature modeling of AlGaN/GaN HEMTs. Solid State Electron..

[28] Hansen P.J., Vaithyanathan V., Wu Y., Mates T., Heikman S., Mishra U.K., York R.A., Schlom J.S., Speck J.S. (2005). Rutile films grown by molecular beam epitaxy on GaN and AlGaN/GaN. J. Vac. Sci. Technol. B.

[29] Yagi S., Shimizu M., Inada M., Yamamoto Y., Piao G., Okumura H., Yano Y., Akutsu N., Ohashi H. (2006). High breakdown voltage AlGaN/GaN MIS–HEMT with SiN and TiO_2_ gate insulator. Solid State Electron..

[30] Yang S., Tang Z., Hua M., Zhang Z., Wei J., Lu Y., Chen K.J. (2020). Investigation of SiN_x_ and AlN Passivation for AlGaN/GaN High-Electron-Mobility Transistors: Role of Interface Traps and Polarization Charges. IEEE J. Electron Devices Soc..

[31] Chen X., Boumaiza S., Wei L. (2019). Self-heating and equivalent channel temperature in short gate length GaN HEMTs. IEEE Trans. Electron Devices.

[32] Islam S., Li Z., Dorgan V.E., Bae M.H., Pop E. (2013). Role of Joule heating on current saturation and transient behavior of graphene transistors. IEEE Electron Device Lett..

[33] Huang S., Liu X., Wei K., Liu G., Wang X., Sun B., Yang X., Shen B., Liu C., Hua M. (2015). O_3_-sourced atomic layer deposition of high quality Al_2_O_3_ gate dielectric for normally-off GaN metal-insulator-semiconductor high-electron-mobility transistors. Appl. Phys. Lett..

[34] Caddemi A., Crupi G., Donato N. (2006). Microwave characterization and modeling of packaged HEMTs by a direct extraction procedure down to 30 K. IEEE Trans. Instrum. Meas..

